# Portal vein wedge resection and patch venoplasty with autologous vein grafts for hepatobiliary–pancreatic cancer

**DOI:** 10.1186/s40792-024-01823-y

**Published:** 2024-01-26

**Authors:** Tadafumi Asaoka, Kenta Furukawa, Manabu Mikamori, Satoshi Hyuga, Tomofumi Ohashi, Iwamoto Kazuya, Yujiro Nakahara, Atsushi Naito, Hidekazu Takahashi, Jeongho Moon, Mitsunobu Imasato, Chu Matsuda, Kazuhiro Nishikawa, Tsunekazu Mizushima

**Affiliations:** Department of Gastroenterological Surgery, Osaka Police Hospital, 10-31 Kitayamacho, Tennouji-Ku, Osaka, 543-0035 Japan

**Keywords:** Perihilar cholangiocarcinoma, Autologous graft, Portal vein reconstruction

## Abstract

**Background:**

Advanced hepatobiliary–pancreatic cancer often invades critical blood vessels, including the portal vein (PV) and hepatic artery. Resection with tumor-free resection margins is crucial to achieving a favorable prognosis in these patients. Herein, we present our cases and surgical techniques for PV wedge resection with patch venoplasty using autologous vein grafts during surgery for pancreatic ductal adenocarcinoma (PDAC) and perihilar cholangiocarcinoma (PhCC).

**Case presentation:**

Case 1: 73-year-old female patient with PDAC; underwent subtotal stomach-preserving pancreatoduodenectomy, with superior mesenteric vein wedge resection and venoplasty with the right gonadal vein. Case 2: 67-year-old male patient with PDAC; underwent distal pancreatectomy and celiac axis resection, with PV wedge resection and venoplasty with the middle colic vein. Case 3: 51-year-old female patient with type IV PhCC; underwent left hepatectomy with caudate lobectomy and bile duct resection, with hilar PV wedge resection and venoplasty with the inferior mesenteric vein (IMV). Case 4: 69-year-old male patient with type IIIA PhCC; underwent right hepatopancreatoduodenectomy, with hilar PV resection and patch venoplasty with the IMV. All patients survived for over 12 months after the surgery, without local recurrence.

**Conclusions:**

PV wedge resection and patch venoplasty is a useful technique for obtaining tumor-free margins in surgeries for hepatobiliary–pancreatic cancer.

## Introduction

Hepatobiliary–pancreatic (HBP) malignancies, such as perihilar cholangiocarcinoma (PhCC) and pancreatic ductal adenocarcinoma (PDAC), can directly invade the portal vein (PV) due to their close proximity to the PV. While complete resection of these tumors with tumor-free resection margins is necessary to achieve a favorable prognosis in patients with these cancers [1–5], vascular resection of the PV increases the risk of postoperative morbidity and mortality [6, 7]. PV resection and reconstruction combined with hepatectomy and pancreatectomy is frequently performed in patients with advanced PhCC and PDAC, because it offers a better chance of long-term survival in selected patients [8]. One of the most common methods of PV resection and reconstruction are segmental PV resection with end-to-end anastomosis or wedge resection of the PV with primary repair. In more advanced cases, interposition grafts have also been considered. However, there are some cases in which reconstruction of the PV using common techniques proves difficult due to the location of the tumor invasion, such as at the perihilar PV bifurcation, superior mesenteric vein (SMV)–jejunal vein (JV) trunk confluence, and the PV–splenic vein (SPV) confluence.

Several unique techniques for patch venoplasty have been reported in cases of living donor liver transplantation (LDLT) to resolve the graft PV size mismatch or allow multiple hepatic vein reconstructions, which have also contributed to advanced surgeries for PV reconstruction in patients with HBP malignancies [9]. Herein, we report our techniques and usefulness for wedge resection of the PV and reconstruction by patch venoplasty using autologous vein grafts in patients with HBP malignancies.

## Case presentation

### Case 1

**Fig. 1:**
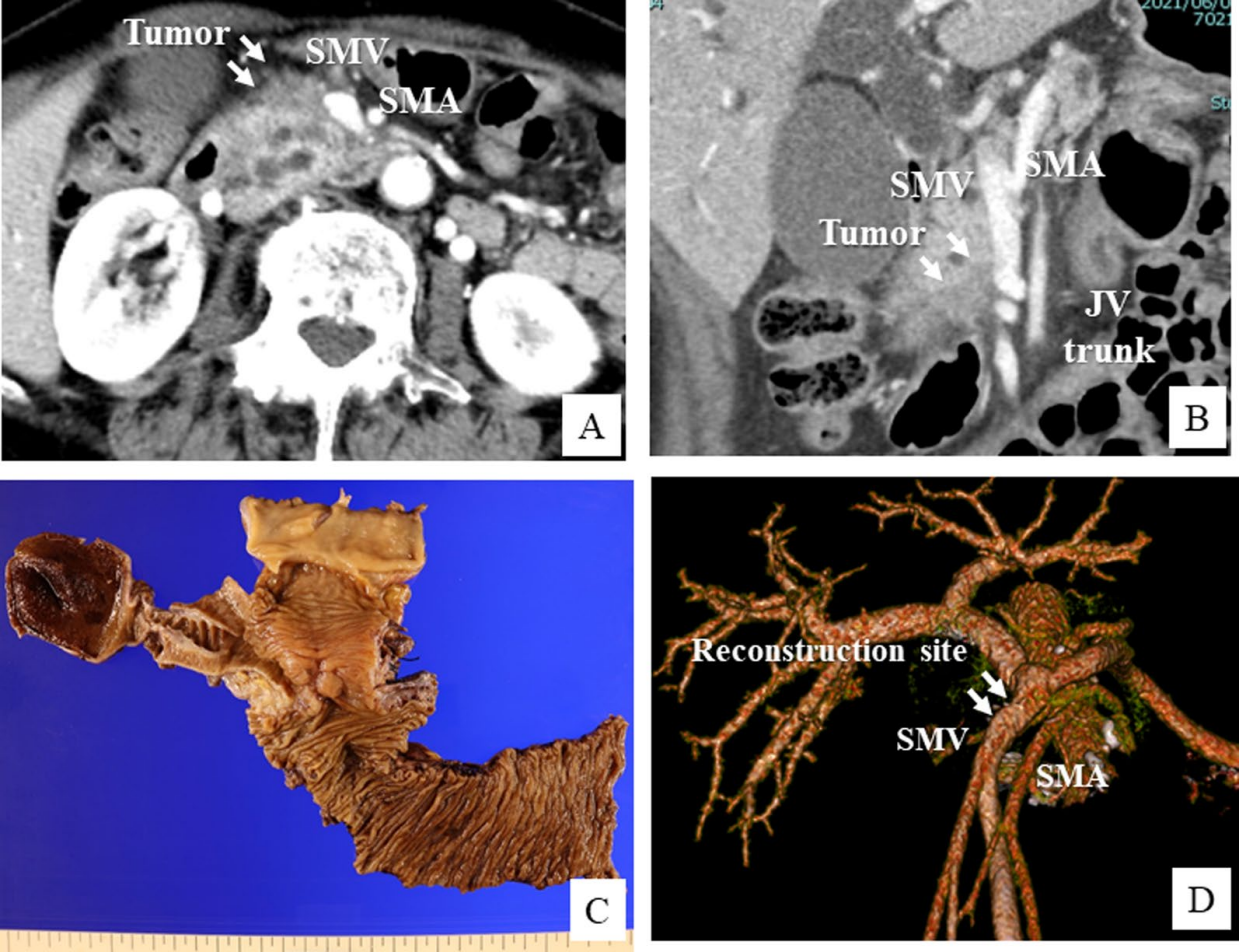
Perioperative findings in Case 1. **A**, **B** Preoperative CT image showing advanced pancreatic head cancer and suspected portal vein invasion (arrow). **C** Macroscopic findings of the surgical specimen after pancreatoduodenectomy. **D** CT image at 6 months after the surgery showing the normal reconstructed site of the superior mesenteric vein (SMV)

**Fig. 2:**
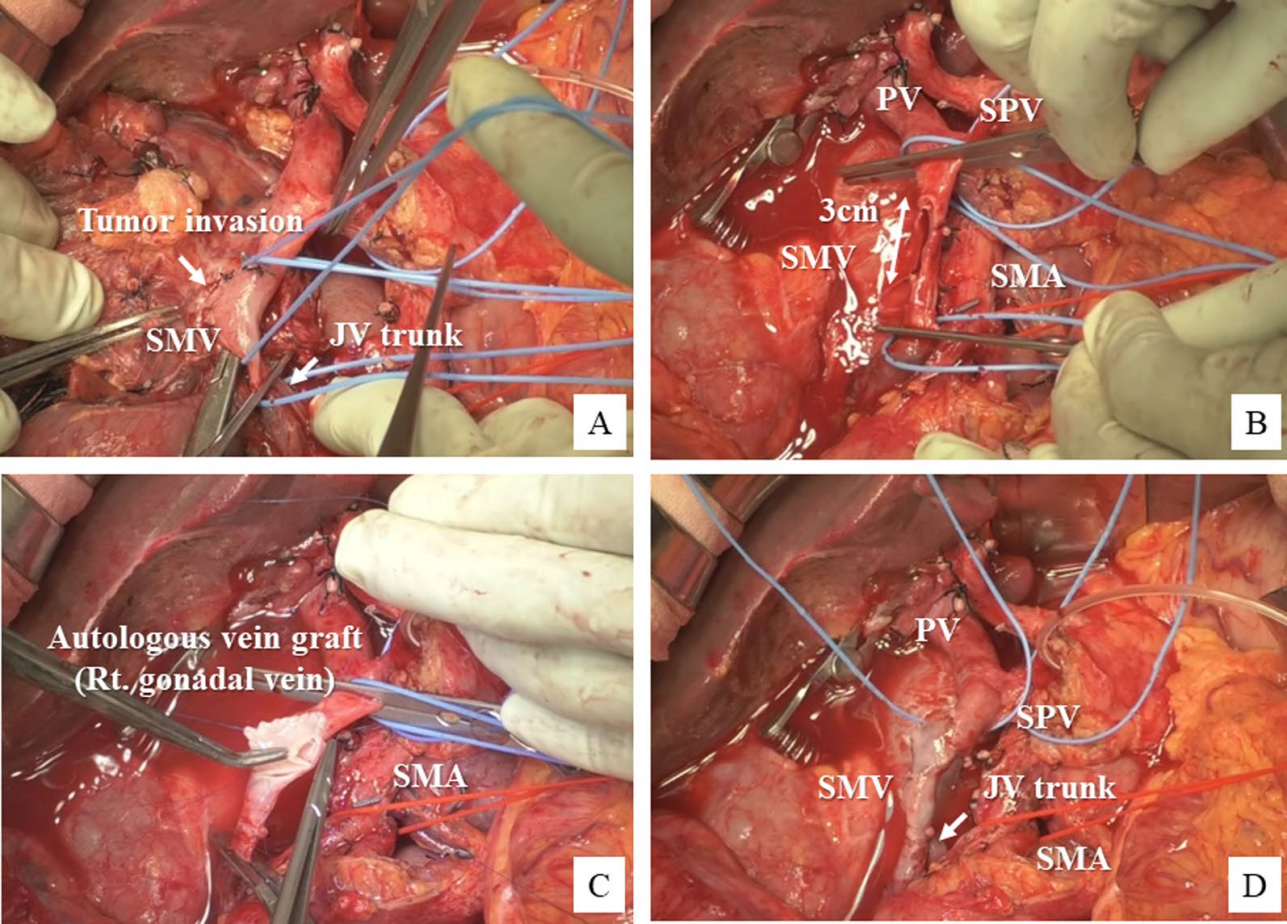
Intraoperative findings in Case 1. **A**, **B** Invaded SMV–jejunal trunk confluence is partially resected over about half of its circumference. **C**, **D** Defect in the SMV wall is reconstructed by patch venoplasty using a right gonadal vein patch graft

A 73-year-old female patient was admitted to our hospital with the diagnosis of advanced pancreatic head cancer. The tumor measured 2.5 cm in diameter and was located close to the SMV–jejunal vein trunk confluence (Fig. 1A, B). Although the tumor was advanced and suspected as having invaded the SMV, neoadjuvant chemotherapy was not performed, because the patient had myasthenia gravis as a comorbidity and required the administration of immunosuppressants. Therefore, we decided to perform pancreatoduodenectomy with SMV reconstruction. During the operation, we found that the tumor had invaded the SMV involving about one-third of its circumference. The SMV–jejunal vein trunk confluence was near the tumor, so that we elected to perform SMV wedge resection with patch venoplasty, to preserve the JV trunk (Fig. 2A, B). After the SMV wall infiltrated by the tumor was elliptically removed, the defect was repaired with an autologous right gonadal vein graft (Fig. 2C, D). Postoperative histopathology revealed that the cancer at the head of the pancreas was an invasive ductal adenocarcinoma measuring 2.8 cm in diameter, and the resected PV segment showed direct tumor invasion (Fig. 1C). Both lymphovascular invasion and perineural invasion were noted and 7 of the resected lymph nodes (LNs) were found to be metastatic. According to the 8th edition of the TNM classification of the American Joint Committee on Cancer (AJCC), the tumor was classified as pT3N1M0, corresponding to stage II B disease. The patient recovered uneventfully and was discharged on postoperative day 18. The patient was diagnosed as having a cervical bone metastasis 8 months after the surgery; however, the patency of the PV in the reconstructed region was still found to be intact (Fig. 1D).

### Case 2

**Fig. 3:**
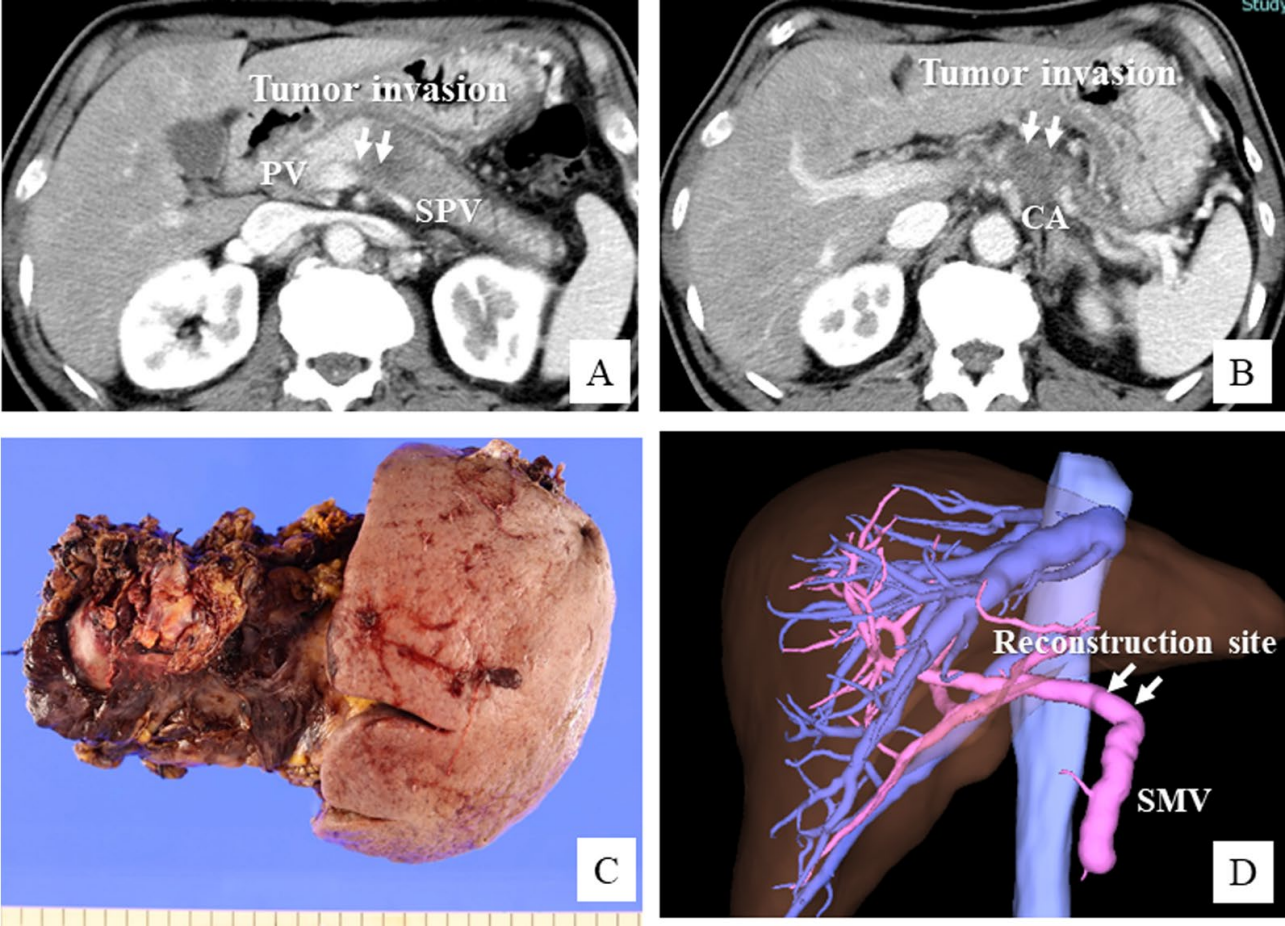
Perioperative findings in Case 2. **A**, **B** Preoperative CT image showing advanced pancreatic cancer with PV invasion at the PV–splenic vein (SPV) confluence. **C** Macroscopic findings of the surgical specimen after distal pancreatectomy with celiac axis resection. **D** CT obtained 6 months after the surgery shows the normal reconstructed site of the superior mesenteric vein

**Fig. 4:**
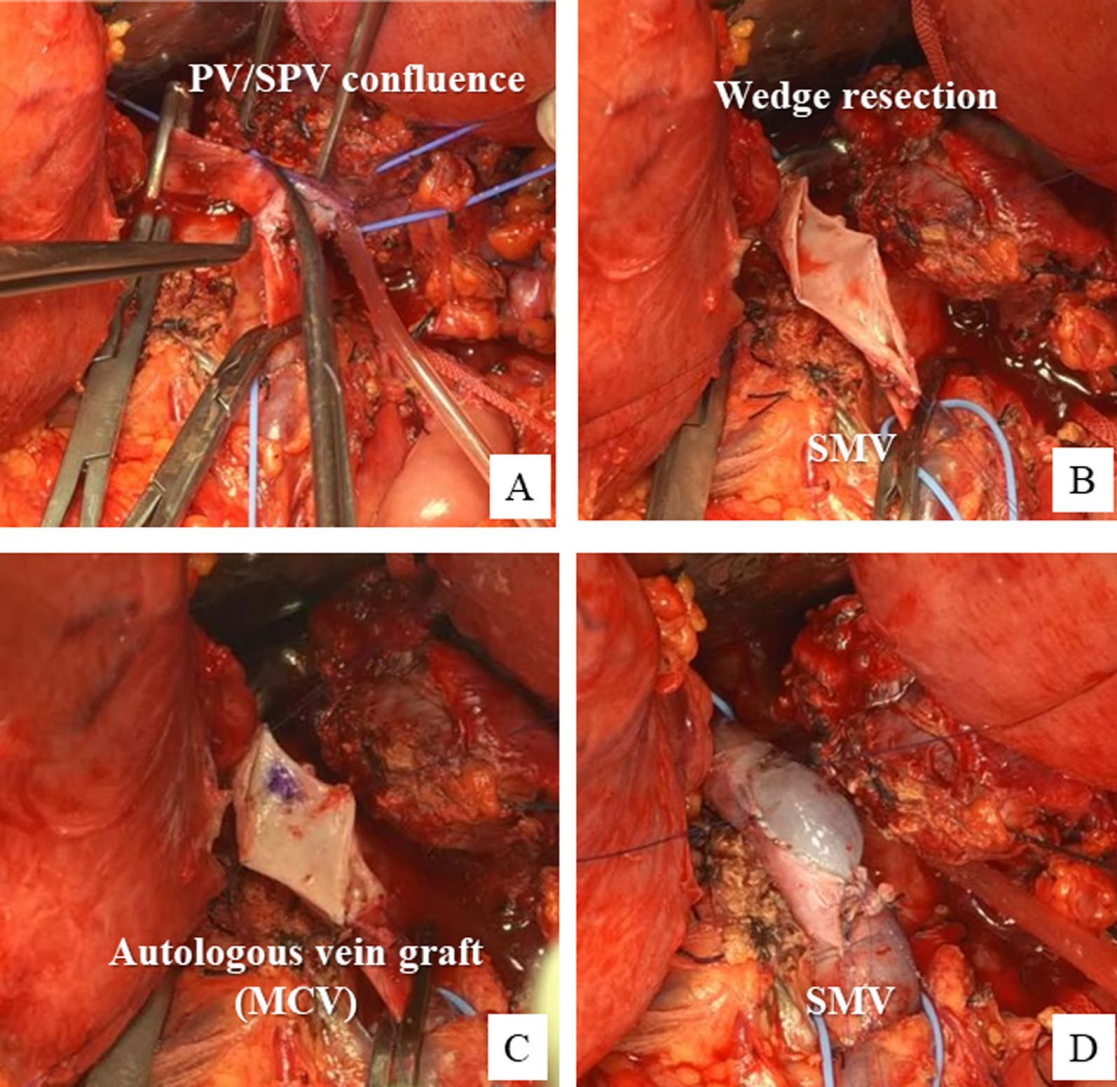
Intraoperative findings in Case 2. **A**, **B** PV–SPV confluence invaded by the tumor is partially resected over about half of its circumference. **C**, **D** Defect in the PV wall is reconstructed with a middle colic vein patch graft

A 67-year-old male patient was admitted to our hospital with the diagnosis of advanced pancreatic body cancer. The tumor invaded the common hepatic artery (CHA) and celiac artery axis (CA), and the paraaortic LNs were enlarged. The patient was classified as having unresectable disease and administered 10 courses of combined chemotherapy with gemcitabine + nab-paclitaxel and 4 courses of modified FOLFIRINOX. After the chemotherapy, the tumor was found to have downsized and the paraaortic LN enlargements had disappeared, although the vascular invasion of the CHA and CA persisted (Fig. 3A, B). We decided to perform surgical resection after further chemoradiotherapy with S-1 and intensity-modulated radiation therapy (IMRT: 60 Gy). During the operation, we found that the tumor had invaded the CHA and PV–SPV confluence (Fig. 4A). Therefore, we performed distal pancreatectomy with celiac axis resection and PV wedge resection. We excised about half the circumference of the PV wall (Fig. 4B), and the defect in the PV wall was repaired with an autologous MCV patch graft (Fig. 4C, D). Postoperative histopathology revealed that the pancreatic tumor was an invasive ductal adenocarcinoma with vascular invasion and perineural invasion (Fig. 3C). One of the 34 resected LNs were found to be metastatic. The clinical stage was Stage III (pT4N1M0) based on the 8th edition of the TNM classification. The extent of the residual carcinoma following neoadjuvant chemoradiation therapy was histologically graded as an Evans grade IIa response. The patient recovered uneventfully from the surgery, and received adjuvant combined chemotherapy with liposomal irinotecan and 5-fluorouracil/leucovorin (nal-IRI + 5-FU/LV) for 6 months. The reconstructed PV site appeared to be intact on the follow-up CT obtained 6 months after the surgery (Fig. 3D). The patient was followed up for 12 months, with no tumor recurrence.

### Case 3

**Fig. 5:**
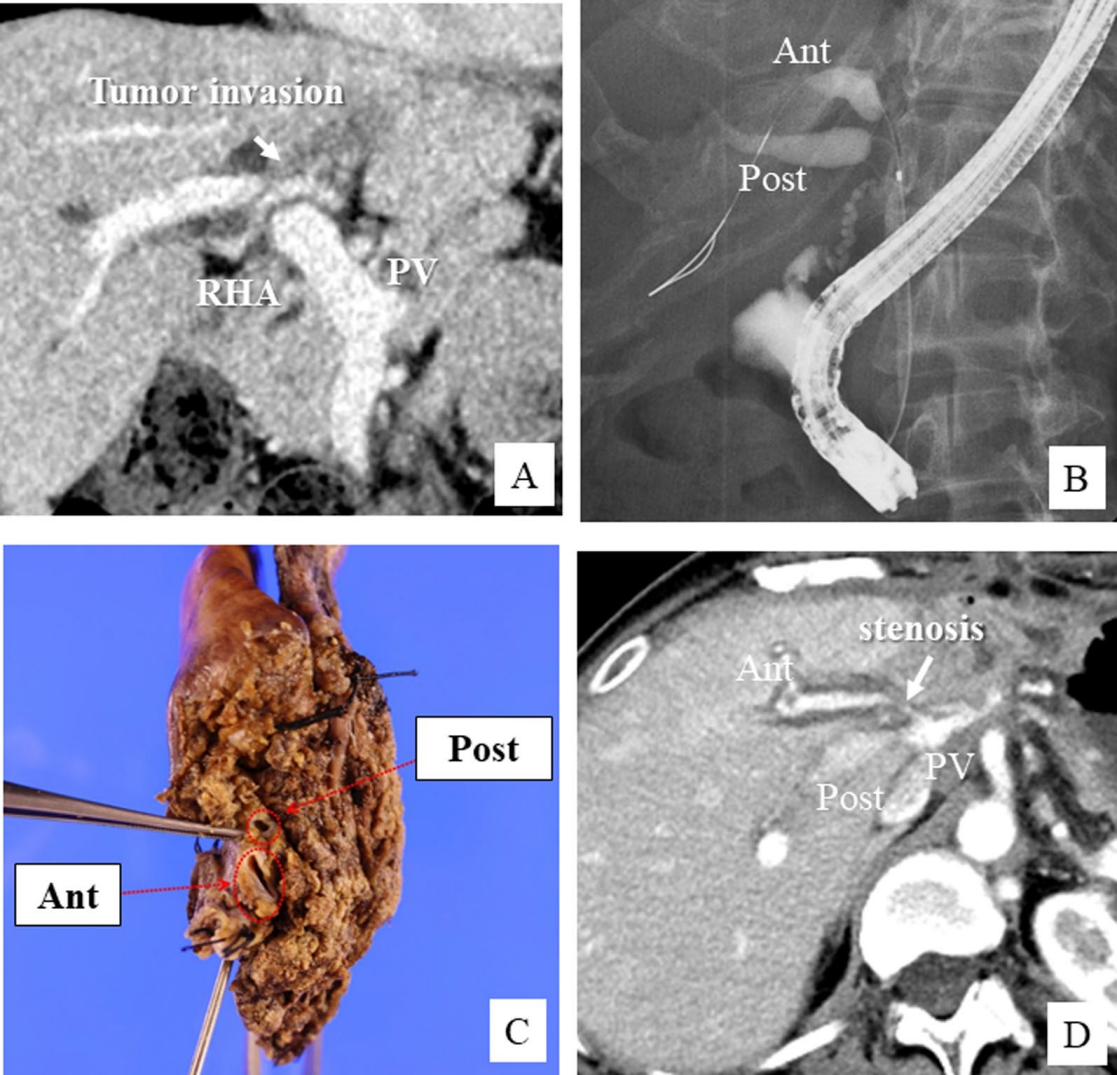
Perioperative findings in Case 3. **A** CT image showing advanced perihilar cholangiocarcinoma invading the hilar PV bifurcation. **B** Endoscopic retrograde cholangiographic image showing Bismuth type IV tumor spread. **C** Macroscopic findings of the surgical specimen after left hemi-hepatectomy with the caudate lobe. **D** CT obtained 6 months after the surgery showed slight stenosis at the site of reconstruction of the anterior PV branch

**Fig. 6:**
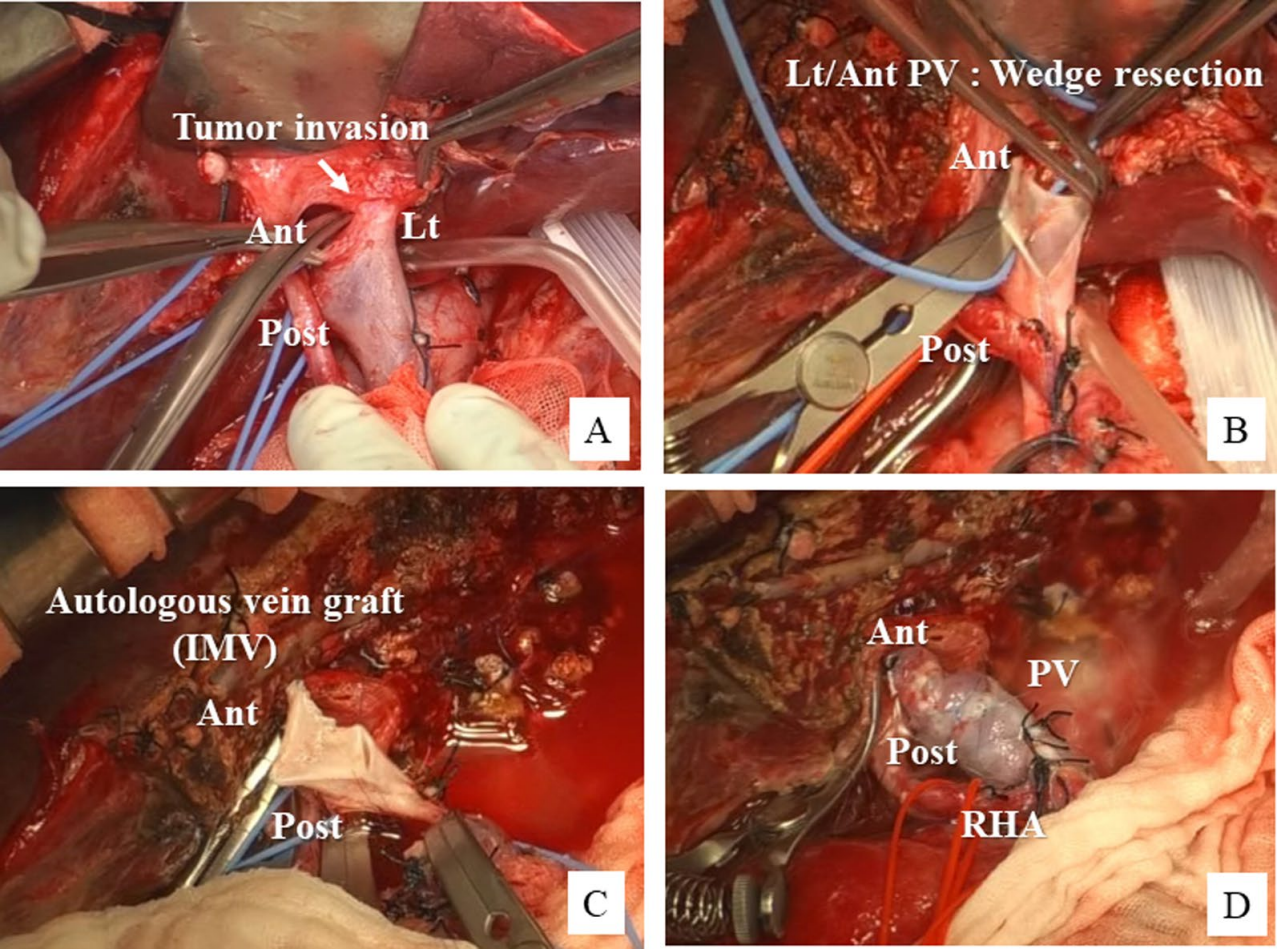
Intraoperative findings in Case 3. **A**, **B** Tumor invading the region of confluence of the right anterior and left PV bifurcation. Left hemi-hepatectomy and partial resection of the right anterior PV wall is performed. **C**, **D** Defect in the PV wall is reconstructed with an inferior mesenteric vein patch graft

A 51-year-old female patient was referred to our institution with the diagnosis of Bismuth–Corlette type IV PhCC. The tumor was found to invade the hilar PV bifurcation from the left PV and enlarged LNs were detected around the hepatoduodenal ligament. The patient was classified as having advanced case and received 6 courses of combined gemcitabine + cisplatin + S-1 (GCS) chemotherapy, the same regimen as that used in the KHBO 1401 trial [10]. After the chemotherapy, the main tumor and LN swelling decreased in size; however, the PV invasion remained (Fig. 5A). We decided to perform left hepatectomy and PV reconstruction. After extended lymphadenectomy and bile duct resection, tumor invasion of the left and anterior segmental branch of the PV was identified (Fig. 6A). We proceeded with liver transection, and after isolation of the main PV and segmental PV branches, we excised the left PV and removed about the half circumference of the anterior segmental PV wall (Fig. 6B). The defect of the PV wall was repaired with an autologous inferior mesenteric vein (IMV) patch graft (Fig. 6C, D). At the last step, a double Roux-en-Y hepaticojejunostomy was performed for biliary reconstruction. Postoperative histopathology identified the tumor as a nodular type of PhCC measuring 15 mm in diameter (Fig. 5C). The depth of tumor invasion extended beyond the bile duct wall, and the left portal vein was involved. Lymphovascular invasion and perineural invasion were present. None of the resected LNs were metastatic. The proximal and distal bile duct margins were tumor-negative. The bile duct tumor was classified as Stage III B ((pT4N0M0), according to the 8th edition of the TNM classification. The patient recovered uneventfully and received adjuvant chemotherapy with S-1 for 6 months. On follow-up CT at 6 months after the surgery, the reconstructed region of the PV appeared to be stenotic (Fig. 5D), but the intrahepatic PV flow was not disturbed. At the last follow-up conducted 16 months after the surgery, the patient was surviving without recurrence.

### Case 4

**Fig. 7:**
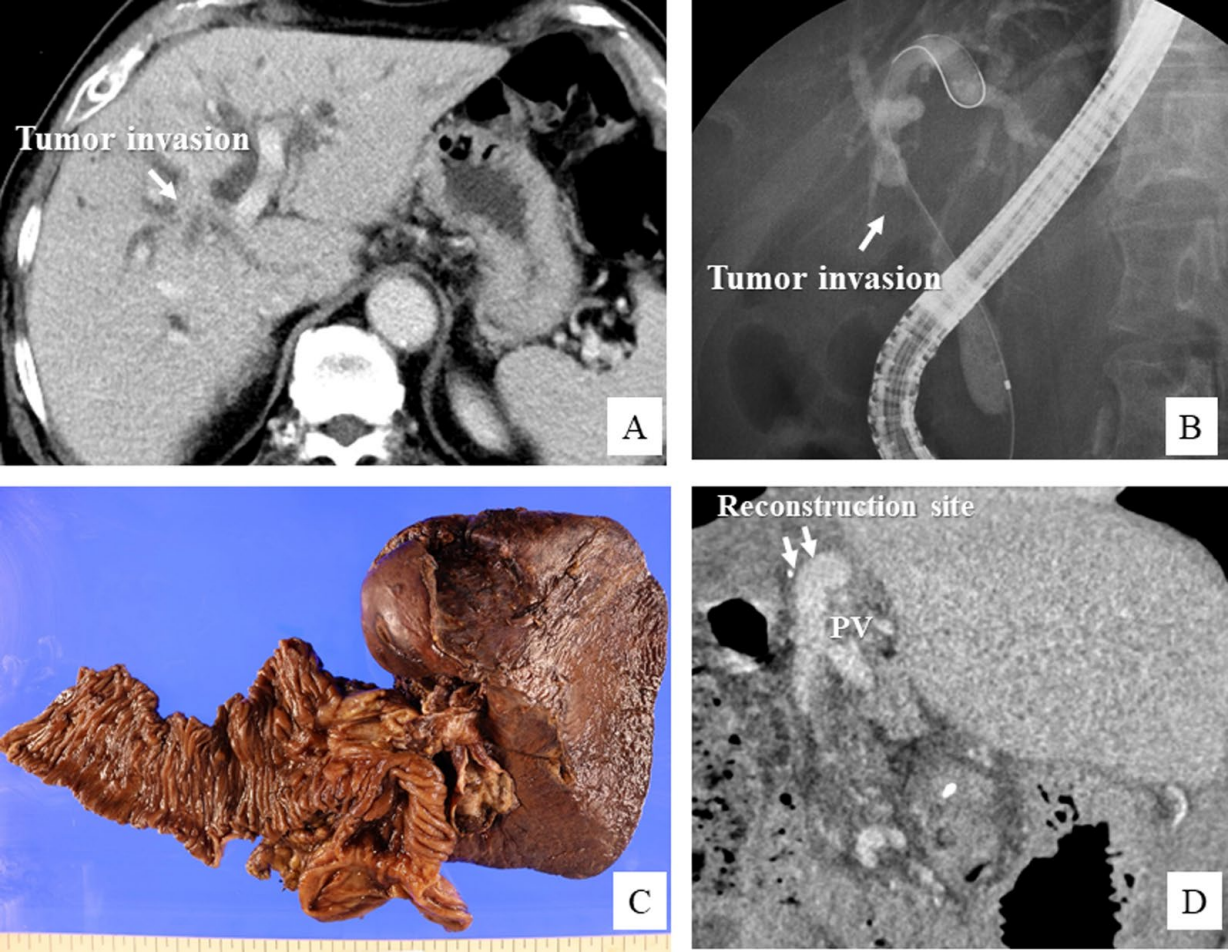
Perioperative findings in Case 4. **A** Preoperative CT image showing advanced perihilar cholangiocarcinoma. **B** Endoscopic retrograde cholangiographic image showing Bismuth type IV tumor spread. **C** Macroscopic findings of surgical specimen after right hemi-hepatectomy with the caudate lobe. **D** CT obtained 3 months after the surgery shows a normal reconstructed site of the PV bifurcation

**Fig. 8:**
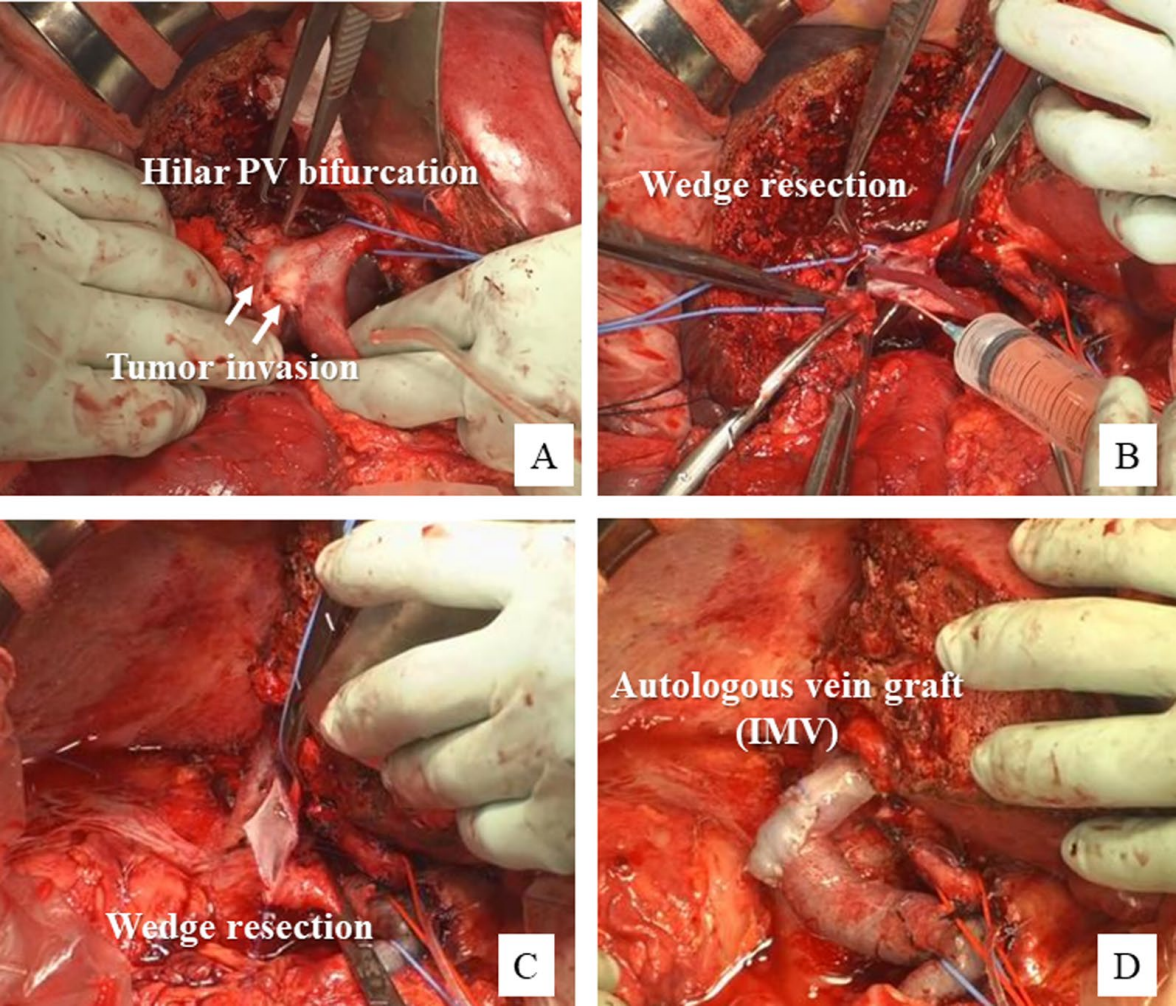
Intraoperative findings in Case 4. **A**, **B** Tumor invading the hilar PV bifurcation. Right hemi-hepatectomy and partial resection of the left PV wall is performed. **C**, **D** Defect in the PV wall is reconstructed with an inferior mesenteric vein patch graft

A 69-year-old male patient was referred to our hospital with the diagnosis of biliary tract cancer. CT showed PhCC, Bismuth–Corlette type III A, with suspected PV invasion (Fig. 7A, B). Furthermore, a mapping biopsy obtained from the distal bile duct during ERCP was positive. CT volumetry showed a residual liver volume ratio of 40%. Percutaneous portal vein embolization (PTPE) was performed before the surgery to reduce the risk of postoperative liver failure. The remnant liver volume ratio increased to 48% at 3 weeks after PTPE. During the operation, the tumor was found to have spread widely, but it still appeared to be resectable. After pancreatoduodenectomy and regional lymph node dissection, tumor invasion of the right PV branch and main PV bifurcation was identified (Fig. 8A). After liver transection, the main portal and hilar portal branches were encircled and the anterior wall of the hilar PV was partially excised (Fig. 8B). The PV wall was reconstructed with an autologous IMV graft (Fig. 8C, D). Roux-en-Y hepaticojejunostomy was performed with the remnant left liver. Postoperative histopathology identified the tumor as a poorly differentiated adenocarcinoma. The tumor extended beyond the bile duct wall into the liver parenchyma and invaded the PV (Fig. 7C). Four of the 35 resected LNs were found to be metastatic. The clinical stage was Stage IV A (pT4N2M0) based on the 8th edition of the TNM classification. The postoperative course was uneventful and no stenosis of the reconstructed PV was detected at follow-up (Fig. 7D). After the surgery, the patient received adjuvant chemotherapy with S-1 and was followed for 12 months without tumor recurrence.

## Discussion

Advanced hepatobiliary–pancreatic cancer sometimes requires combined portal vein resection and reconstruction. However, there are various reconstruction methods depending on the extent and site of tumor invasion of the PV. In general, wedge resection and primary suture are acceptable in cases with tumor involvement up to 1/4 of the PV circumference. If the tumor invasion involves up to 1/2 of the circumference, wedge resection with patch venoplasty is preferable. In cases with tumor invasion extending beyond half of the vein circumference, segmental resection and end-to-end anastomosis or complete replacement with an interposition graft are often selected.

In pancreatic head cancer patients with PV invasion, when the tumor invasion is observed at the confluence of the PV and SMV or at that of the SMV and JV trunk, PV segmental resection and end-to-end anastomosis can be facilitated by sacrificing the SPV and the JV trunk. However, non-reconstruction of the SPV may lead to left-sided portal hypertension and sacrifice of JV trunk can lead to congestion in Roux-en-Y hepaticojejunostomy [11]. Especially, some previous reports have demonstrated that the lack of the SPV can lead to complications, such as stomach congestion and splenomegaly, and, furthermore, lead to bleeding gastroesophageal varices [12–14]. Recently, the usefulness of SPV–left renal vein reconstruction has been reported for the management of left-sided portal hypertension [15]. However, there has been no consensus so far on whether the SPV should be reconstructed or not. If the tumor invasion involves less than half of the circumference, patch venoplasty with an autologous vein graft is a good option, because it is not necessary to sacrifice the SPV or the JV trunk.

In addition, in distal pancreatectomy with combined resection of the PV segment exceeding a length of 3 cm, end-to-end anastomosis of the PV becomes difficult and an interposition graft will be required in some cases [16], because the morbidity of the PV is poor in distal pancreatectomy unlike in pancreatoduodenectomy. According to a previous report, resection of a PV segment measuring ≥ 31 mm and direct end-to-end anastomosis was associated with the development of severe anastomotic stenosis, and the authors recommend venous autografting in such cases [17]. If the tumor PV invasion involves less than half of the circumference, PV wedge resection with primary closure or patch venoplasty could be a relatively easy option. However, in a situation, where primary closure carries a risk of stenosis or deformation of the PV, patch graft venoplasty may be preferable.

In some cases of PhCC with PV invasion, PV reconstruction is difficult due to branch variations and caliber changes between the primary branch and main trunk of the PV. In particular, it is difficult to perform segmental resection and end-to-end anastomosis in cases with a PV variant of the trifurcation type [18], such as in case No. 3; in such cases, PV wedge resection with patch venoplasty would appear to be useful.

Vascular grafts for patch venoplasty are broadly classified into synthetic and biological grafts, but biological graft are preferable, because synthetic grafts are more thrombogenic; the early and overall graft thrombosis rate were reported to be 7.5% and 22.2% for synthetic grafts, and 5.6% and 11.7% for autologous vein grafts [19]. Synthetic grafts need long-term anticoagulation; furthermore, they cannot be used in contaminated surgeries due to the risk of graft infection. Among biological grafts, in recent years, tissue banks have provided cryopreserved vein grafts, but they are still not easily available in most of the centers. The most desirable grafts still remain autologous vein grafts, such as gonadal vein, IMV and MCV grafts, which can be harvested minimally invasively in the same surgical field. The great saphenous vein is another option, but it is invasive in that it requires a new incision for graft harvesting. Recanalized umbilical vein is also an excellent vascular graft that is easy to harvest and can be used for multiple vascular repairs [20, 21]. However, not all umbilical veins necessarily have vascular endothelium [22]; therefore, anticoagulation may be necessary to prevent thrombosis.

If the defect is large or the PV has to be totally replaced, it is better to harvest the left renal vein, internal jugular vein, or external iliac vein. A bovine pericardial patch, which is a commercially available xenograft, is also one of options in such cases [23]. If it is difficult to obtain a vascular graft or if revascularization is accidentally required, an autologous peritoneo-fascial patch would seem to be a safe and versatile option for venous vascular reconstruction, especially in operations with a high risk of contamination [24]. However, since both bovine pericardial patch and peritoneo-fascial grafts lack a vascular endothelium, perioperative anticoagulant therapy is required to prevent thrombosis.

The crucial point in patch venoplasty is to use a large vein patch graft sufficient to avoid stenosis due to atrophic degeneration and gastrointestinal compression [25, 26]. Our case No. 3 showed severe stenosis on follow-up CT obtained 3 months later after surgery, even though the portal venous flow was well-maintained in the perioperative period; the stenosis was probably a result of compression by the hepaticojejunostomy crossing the PV reconstruction site. If PV stenosis is observed in the perioperative period, anticoagulation to prevent PV thrombosis and PV stenting should be considered in cases with liver dysfunction and portal hypertension [27, 28].

Although one of our four cases reported herein developed bone metastasis, curative resection could be performed in all four cases, and all four patients survived for at least 1 year after the procedure with no local recurrence at the site of PV reconstruction (Table 1).

**Table 1:** Summary of our patch venoplasty series

Case	Age	Gender	Primary disease	Operation	Tumor invasion	Vein graft	PV patency	DFS (month)	OS (month)
1	73	Female	PDAC	SSPPD	SMV–JV trunk confluence	Right gonadal vein	No stenosis	Bone meta (8 m)	Alive (12 m)
2	67	Male	PDAC	DP–CAR	PV–SPV confluence	MCV	No stenosis	No recurrence (12 m)	Alive (12 m)
3	51	Female	PhCC	Left hemihepatectomy	Hilar PV bifurcation	IMV	Stenosis	No recurrence (16 m)	Alive (16 m)
4	69	Male	PhCC	Right-HPD	Hilar PV bifurcation	IMV	No stenosis	No recurrence (12 m)	Alive (12 m)

*PDAC* pancreatic ductal adenocarcinoma, *PhCC* perihilar cholangiocarcinoma, *SSPPD* subtotal stomach-preserving pancreatoduodenectomy, *DP–CAR* distal pancreatectomy with celiac axis resection, *HPD* hepatopancreatoduodenectomy, *SMV* superior mesenteric vein, *JV trunk* jejunal vein trunk, *PV* portal vein, *SPV* splenic vein, *IMV* inferior mesenteric vein, *MCV* middle colic vein, *DFS* disease-free survival, *OS* overall survival

Actually, there are various methods for PV reconstruction, but patch venoplasty with autologous vein grafts is effective in situations, where it is difficult to perform end-to-end anastomosis. Especially, in our series, we indicated the most useful situations such as cases with a PV variant of the trifurcation type or distal pancreatectomy with PV reconstruction.

## Conclusion

Portal vein wedge resection with patch venoplasty is a feasible and useful technique to reduce the risk of perioperative morbidity and achieve resection with tumor-free margins in patients with hepatobiliary–pancreatic cancer.

## Data Availability

Not applicable.

## Abbreviations

PhCC: Perihilar cholangiocarcinoma
PDAC: Pancreatic ductal adenocarcinoma
PV: Portal vein
SMV: Superior mesenteric vein
IMV: Inferior mesenteric vein
SPV: Splenic vein
CHA: Common hepatic artery
CA: Celiac artery
LN: Lymph node
